# Brèche ostéoméningée: complication inhabituelle d'un prolactinome traité médicalement

**DOI:** 10.11604/pamj.2015.21.108.6932

**Published:** 2015-06-10

**Authors:** Amal Touiti, Ghizlane El Mghari

**Affiliations:** 1 Service d'Endocrinologie, Diabétologie et Maladies Métaboliques, Hôpital Arrazi, CHU Mohammed VI, Laboratoire de Recherche de Pneumo-Cardio-Immunopathologie et Métabolisme (PCIM), Faculté de Médecine et de Pharmacie de Marrakech, Université Cadi Ayad, Marrakech, Maroc

**Keywords:** Brèche ostéoméningée, prolactinome, syndrome tumoral, Osteomeningea breach, prolactinoma, tumor syndrome

## Image en medicine

Nous rapportons une observation d'une patiente âgée de 19 ans, suivie depuis septembre 2014 pour un macroaprolactinome révélé par un syndrome tumoral avec exophtalmie droite avec à l'IRM un volumineux processus lésionnel intra et extra sellaire infiltrant les sinus caverneux, les artères carotides, le cavum avec extension endoorbitaire droite responsable d'une exophtalmie grade II (A). Le bilan hormonal a mis en évidence une hyperprolactinémie, un déficit thyréotrope et corticotrope. La patiente a été mise alors sous Cabergoline 2 mg/ semaine. L’évolution a été marquée par la survenue d'un tableau d'apoplexie hypophysaire et une rhinorrhée. La TDM cérébrale a mis en évidence une lyse de l’étage moyen de la base du crane avec érosion du dorsum et du plancher sellaire créant une communication avec le sinus sphénoïdal compliqué de pneumencéphalie et de pneumorbite droit (B). L'IRM hypophysaire a montré la présence d'une cavité hydroaérique sellaire et suprasellaire avec résidu tumoral en sa périphérie infiltrant le sinus caverneux homolatéral. (C, D). Le traitement a consisté à un traitement médical par inhibiteur de l'anhydrase carbonique avec une régression de la rhinorrhée. Cette observation souligne la possibilité de survenue de rhinorrhée par fistule au cours de l’évolution des macroprolactinomes traités médicalement. C'est une complication pouvant être grave du fait du risque important de méningite.

**Figure 1:**
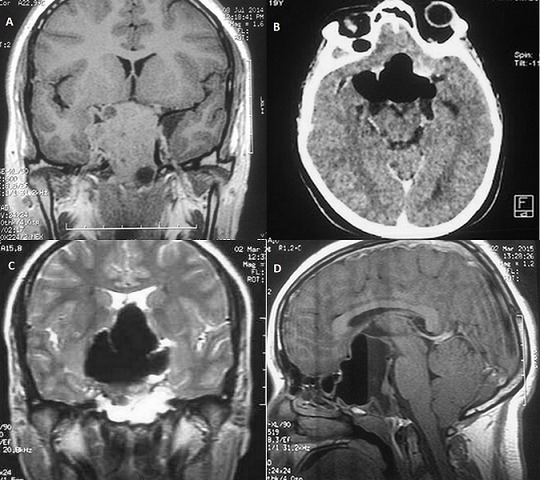
(A) IRM Hypothalamique initiale: coupe coronale: volumineux processus lésionnel intra et extra sellaire infiltrant les sinus caverneux et les artères carotides; (B) TDM cérébrale: coupe axiale: pneumencephalie; (C) IRM cérébrale de contrôle: coupe coronale: cavité hydroaérique sellaire et suprasellaire avec résidu tumoral. (D) Coupe sagittale

